# The causal effect of schizophrenia on fractures and bone mineral density: a comprehensive two-sample Mendelian randomization study of European ancestry

**DOI:** 10.1186/s12888-023-05196-8

**Published:** 2023-09-25

**Authors:** Ningning Jia, Lin Dong, Qingxing Lu, Xinwei Li, Mengdi Jin, Xuyuan Yin, Zhenhua Zhu, Qiufang Jia, Caifang Ji, Li Hui, Qiong Yu

**Affiliations:** 1 Department of Epidemiology and Biostatistics, School of Public Health, Jilin University, Changchun, 130021 China; 2 Research Center of Biological Psychiatry, Suzhou Guangji Hospital, Suzhou Medical College of Soochow University, No. 11 Guangqian Road, Suzhou, Jiangsu 215137 PR China

**Keywords:** Schizophrenia, Fractures, Bone mineral density, Causality, Mendelian randomization

## Abstract

**Background:**

Schizophrenia was clinically documented to co-occur with fractures and aberrant bone mineral density (BMD), but the potential causal relationship remained unclear. This study aimed to test the causal effects between schizophrenia and fractures as well as aberrant BMD by conducting Mendelian randomization (MR) analyses.

**Methods:**

Two-sample MR was utilized, based on instrumental variables from large genome-wide association studies (GWAS) of schizophrenia as exposure, to identify the causal association of schizophrenia with mixed fractures, fractures at different body sites (including skull and facial bones, shoulder and upper arm, wrist and hand, and femur) and BMDs of forearm (FA), femoral neck (FN), lumbar spine (LS) and estimated BMD (eBMD). Multivariable Mendelian randomization (MVMR) analysis was performed to minimize the confounding effect of body mass index (BMI).

**Results:**

Result from inverse variance weighting (IVW) method provided evidence schizophrenia increased the risk of fractures of skull and facial bones [odds ratio (OR) = 1.0006, 95% confidence interval (CI): 1.0003 to 1.0010] and femur [OR =1.0007, 95% CI: 1.0003 to 1.0011], whereas, decreased the level of eBMD [β (95%CI): -0.013 (-0.021, -0.004)]. These causal effects still existed after adjusting for BMI. Sensitivity analyses showed similar results. However, no causal effect of schizophrenia on fracture or BMD in other parts was detected.

**Conclusion:**

The current finding confirmed that schizophrenia was causally associated with the fractures of skull, face and femur as well as eBMD, which might remind psychiatrists to pay close attention to the fracture risk in schizophrenic patients when formulating their treatment strategies.

**Supplementary Information:**

The online version contains supplementary material available at 10.1186/s12888-023-05196-8.

## Introduction

Schizophrenia is a severe, complex and neuropsychiatric disorder with marked functional impairment posing a considerable societal burden [1, 2], affecting about 1% of the world’s population. Compared with the general population, patients with schizophrenia experience poor general health outcomes, including increased risks of osteoporosis which is characterized by abnormally low bone mineral density (BMD), and fracture, especially hip fracture [3–5]. However, the pathogenesis of osteoporosis and fracture in patients with schizophrenia are not clearly defined. Previous studies have reported that hyperprolactinemia caused by the long-term use of antipsychotics accelerates bone turnover, which results in hypothalamic-pituitary-gonadal axis mediating osteopenia so as to increase the risk of fracture [6, 7]. In patients with schizophrenia, other risk factors for osteoporosis and fractures such as lack of physical activity, diabetes, smoking, excessive drinking and vitamin D deficiency are more prevalent [8, 9], which may further contribute to the occurrence and development of schizophrenia. Therefore, the causal association of schizophrenia with fracture and osteoporosis should be worth investigating.

Thus far, the evidence of the relationship between schizophrenia and fracture mainly comes from the relevant research or clinical observation, and the confusion triggered by intermediary factors and possible reverse causal relationship hinders the exploration of causal effect. While conducting a randomized controlled trial (RCT) to determine the causal factors is not feasible, Mendelian Randomization (MR), a widely used method of causal inference, is applied to infer the causality of risk factor “exposures” to disease “outcomes” in case to the circumvent confounding bias and reverse causation [10]. Recent large-scale genome-wide association studies (GWAS) have identified the multiple genetic variants associated with complex human traits or diseases, including schizophrenia [11–13], which implements two-sample MR by using the variants as the instrumental variables (IVs) with increased statistical power to detect the potential causal association of schizophrenia with other traits.

However, few studies investigated the causal association of schizophrenia with fractures and BMD in the population coming from European ancestry. Thus, we performed a two-sample MR, based on genome-wide GWAS summary statistics, to investigate the causal effect of schizophrenia on fractures and BMD. Given the various incidence and severity of different fracture sites in patients with psychiatric disorders [4], we further explored the causal effect of schizophrenia on site-specific fractures and BMD. In addition, several studies verified that low BMI was associated with increased fracture risk and low BMD [14, 15]. A recent MR study based on GWAS also demonstrated a causal relationship between BMI and estimated BMD (eBMD) [16]. Consequently, we performed a Multivariable Mendelian randomization (MVMR) analysis to eliminate the effect of BMI confounder.

## Methods

### Study design

**Fig. 1:**
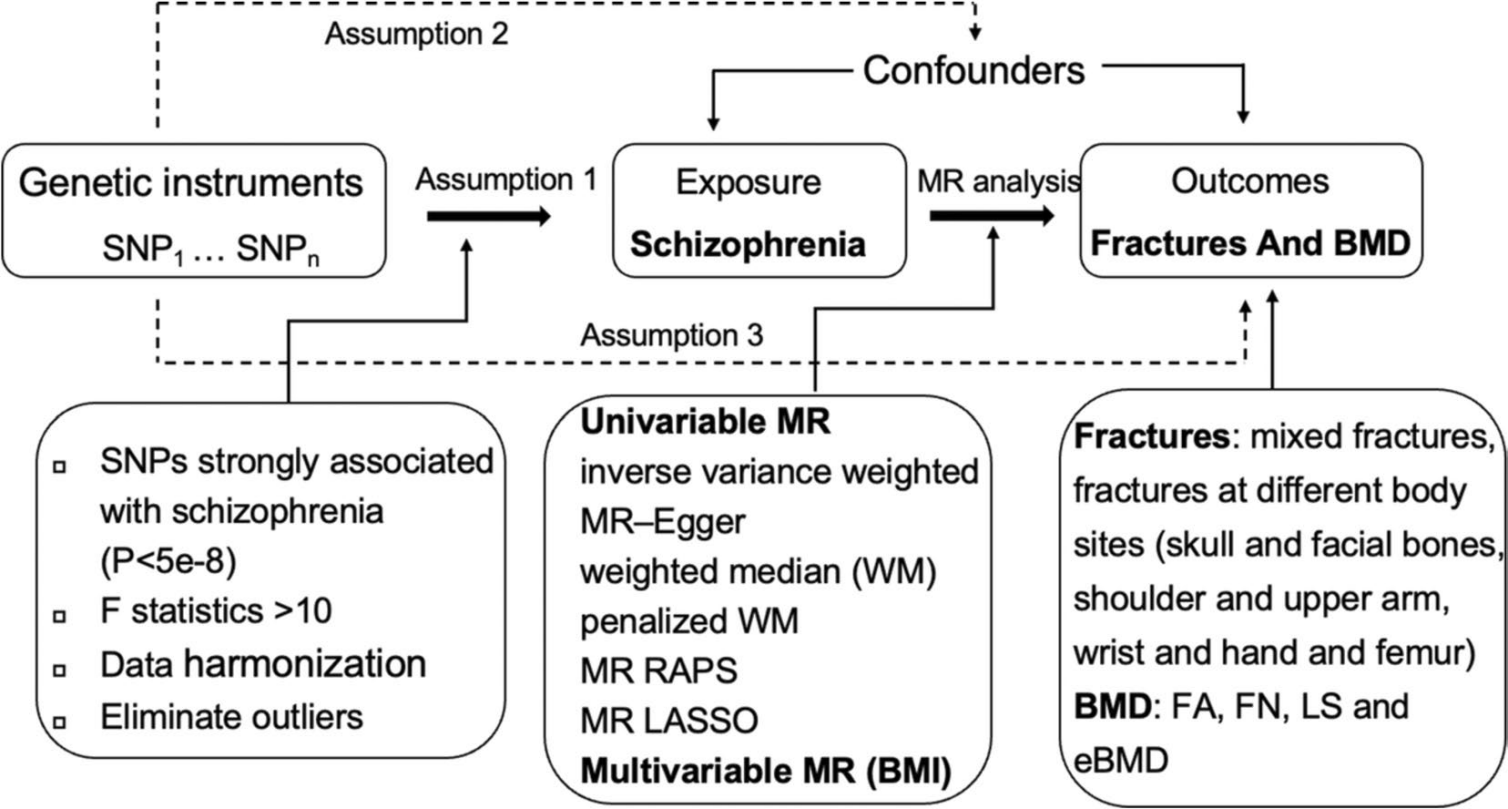
Assumptions of Mendelian randomization (MR) analysis and design of the study. MR analysis was conducted based on three hypotheses that the genetic variants in the instrument must (1) associate robustly with schizophrenia; (2) be independent of confounders; and (3) not directly affect the outcome. According to MR assumptions, solid lines are hypothesized to exist whereas dashed lines are theorized to be insignificant

The schematic of this study was shown in Fig. 1. Briefly, we performed a two-sample MR to explore the causal effect of schizophrenia on the site-specific fractures and BMD, and MVMR analysis was further conducted to eliminate the effect of BMI confounder.

### Data resource

Summarized data only for the European population were adopted to remove the bias of ethnic heterogeneity. The GWAS summary data associated with schizophrenia was derived from Psychiatric Genomics Consortium (PGC, https://figshare.com/articles/dataset/scz2022/19426775). The GWAS meta-analysis included up to 7,585,078 SNPs with MAF ≥ 1% in 175,799 individuals that 74.3% of whom were European (53,386 schizophrenia and 77,258 controls), and the SNP-based heritability was estimated to be 0.24 in European sample [17].

Mixed fracture (including fractures in all parts) GWAS summary data consisting of 53,184 cases and 373,611 controls of white British participants were downloaded from Genetic Factors for Osteoporosis (GEFOS) consortium (http://www.gefos.org) [18]. However, this GWAS did not include site-specific fractures. We further downloaded the GWAS statistic from UK Biobank (http://www.nealelab.is/uk-biobank) for fracture sites according to the International Classification of diseases (ICD), including skull and facial bones, shoulder and upper arm, wrist and hand and femur. Compared with other body sites, loss of bone mass in forearm (FA), femoral neck (FN), lumbar spine (LS) are prone to osteoporotic fractures [19]. The BMD GWAS summary data of FA (*n* = 8,143), FN (*n* = 33,297) and LS (*n* = 32,735) were obtained from GEFOS [20], which were all measured by the dual-energy X-ray absorptiometry (DXA) machines. Each SNP with a minor allele frequency (MAF) > 0.5% was tested for its effect on BMD after adjusting for sex, age and weight. Both ultrasound and DXA-derived BMD are strongly associated with the fracture risk, so we also captured an eBMD GWAS of heel quantitative ultrasound (HL) in 426,824 individuals, identifying 518 genome-wide significant loci (301 novels) and explaining 20% of its variance [18].

A recent GWAS study identified 951 near-independent signals associated with BMI explaining 6.0% of the variance of BMI in 681,275 European participants [21]. The GWAS summary data on BMI was acquired from the Genetic Investigation of ANthropometric Traits (GIANT) consortium (https://portals.broadinstitute.org/collaboration/giant/index.php/GIANT_consortium_data_files). Details of data sources are presented in Table 1.

**Table 1:** Description of all GWAS summaries performed for Mendelian randomization

Traits	Year	Authors	Population	Consortium	Sample size	Number of SNPs
Schizophrenia	2022	Vassily et al.	European	PGC	130,644	7,659,767
Mixed fracture	2018	John et al.	European	GEFOS	416,795	13,977,204
Fracture of skull and facial bones	2018	/	European	Neale Lab	361,194	9,812,773
Fracture of shoulder and upper arm	2018	/	European	Neale Lab	361,194	10,299,263
Fracture of wrist and hand	2018	/	European	Neale Lab	361,194	10,256,723
Fracture of femur	2018	/	European	Neale Lab	361,194	10,334,675
eBMD	2018	John et al.	European	Neale Lab	426,824	13,737,936
Femoral neck BMD	2015	Zheng et al.	European	GEFOS	32,735	10,586,900
Lumbar spine BMD	2015	Zheng et al.	European	GEFOS	28,498	10,582,867
Forearm BMD	2015	Zheng et al.	European	GEFOS	8,143	9,955,366
Body Mass Index	2018	Loic et al.	European	GIANT	681,275	2,336,260

### Genetic instrumental variables

We selected the SNPs strongly associated with schizophrenia at the level of genome-wide significance (*P* < 5e-8). To validate all the IVs for exposure were not in linkage disequilibrium (LD), we performed the clumping process (R^2^ < 0.001, window size = 1000 kb) with the European population from the 1000 genomes project to estimate LD between SNPs. In addition, we removed the SNPs with MAF < 0.01, 189 independent SNPs were obtained at this step. We calculated the F statistic (F= (β/se)^2^) of each SNP to test for the presence of weak IVs according to the assumptions of MR analysis. For these IVs, all F statistics were above 10 (ranging from 29.5 to 175.3) which were listed in Supplement Table S1, excluding the possibility of weak IVs. Then, we excluded the SNPs which had link to any outcome (*P* < 5e-8) to extract the above-selected SNPs. In the harmonizing process, ambiguous SNPs with non-concordant alleles (e.g., A/G vs. A/C) were corrected, and palindromic SNPs with an ambiguous strand (i.e., A/T or G/C) were directly excluded to ensure that the effect of each SNP on both the exposure and the outcomes corresponded to the same allele. We implemented the MR PRESSO and Radial Regression to identify and eliminate outliers toward diminishing heterogeneity. These rigorously screened SNPs served as the final IVs for subsequent two-sample MR analyses.

### Two-sample MR

Several robust approaches were proposed in case of pleiotropy or weak instrument bias, which involved inverse variance weighting (IVW), MR–Egger, weighted median (WM), penalized weighted median (penalized WM), robust-adjusted profile score (MR RAPS) and MR LASSO. IVW was deemed as the primary analysis, which utilized an inverse-variance weighted formula to estimate the combined causal effects and minimize the variance of the weighted average simultaneously [22]. Based on the assumption that the pleiotropic associations were independent, MR-Egger performed a weighted linear regression of the outcome coefficients on the exposure coefficients. The weighted median estimator had similar efficiency to the IVW method [23], as an adjunct to IVW. In the case of IV heterogeneity, penalized WM and MR LASSO were robust [24]. MR RAPS provided an overall estimator that was robust to systematic and idiosyncratic pleiotropy by using robust adjusted profile scores to correct for pleiotropy [25]. Moreover, we applied MR Steiger to detect the causal direction of extracted IVs on exposure and outcomes, in which a “TRUE” result suggested causality in the expected direction, otherwise indicating the opposite direction. R^2^ values were calculated as the sum of 2* EAF* (1 - EAF) * β^2^ and powers were calculated using https://sb452.shinyapps.io/power/.

### Pleiotropy and sensitivity analysis

MR-Egger was performed to access the potential pleiotropic effects based on the intercept [26], with interceptions close to zero, indicating no horizontal pleiotropy. We also conducted the MR-PRESSO test to identify horizontal pleiotropic outliers in multi-instrument summary-level MR and the global test indicated if pleiotropy existed [27]. The heterogeneities were quantified by Cochran Q statistic for IVW and MR-Egger. MR PRESSO and Radial Regression [28] to identify and eliminate outliers toward diminishing heterogeneity. Additionally, a “leave-one-out” sensitivity analysis was applied where the MR has performed again but leaving out each SNP in turn to identify potentially influential SNPs.

### Multivariable mendelian randomization

We searched traits related to schizophrenia-associated SNPs in the PhenoScanner V2 database (http://www.phenoscanner.medschl.cam.ac.uk), with the aim of covering the confounding factors associated with the IVs. BMI was the most likely potential confounder for our selection of IVs, additionally, BMI was well established with regard to fracture and BMD. We performed MVMR analysis considering schizophrenia and BMI commonly as exposure to obtain the independent effect of schizophrenia on fracture and BMD derived by discharging BMI confounding.

### Statistical analyses

All of these analyses were implemented in R 4.1.1 version by using packages of “TwoSampleMR”, “MR-PRESSO”, “RadialMR”, “MendelianRandomization” and “Knitr”. *P* value < 0.05 was considered statistically significant.

## Results

### Two-sample MR for the causal effect of schizophrenia on fracture

**Fig. 2:**
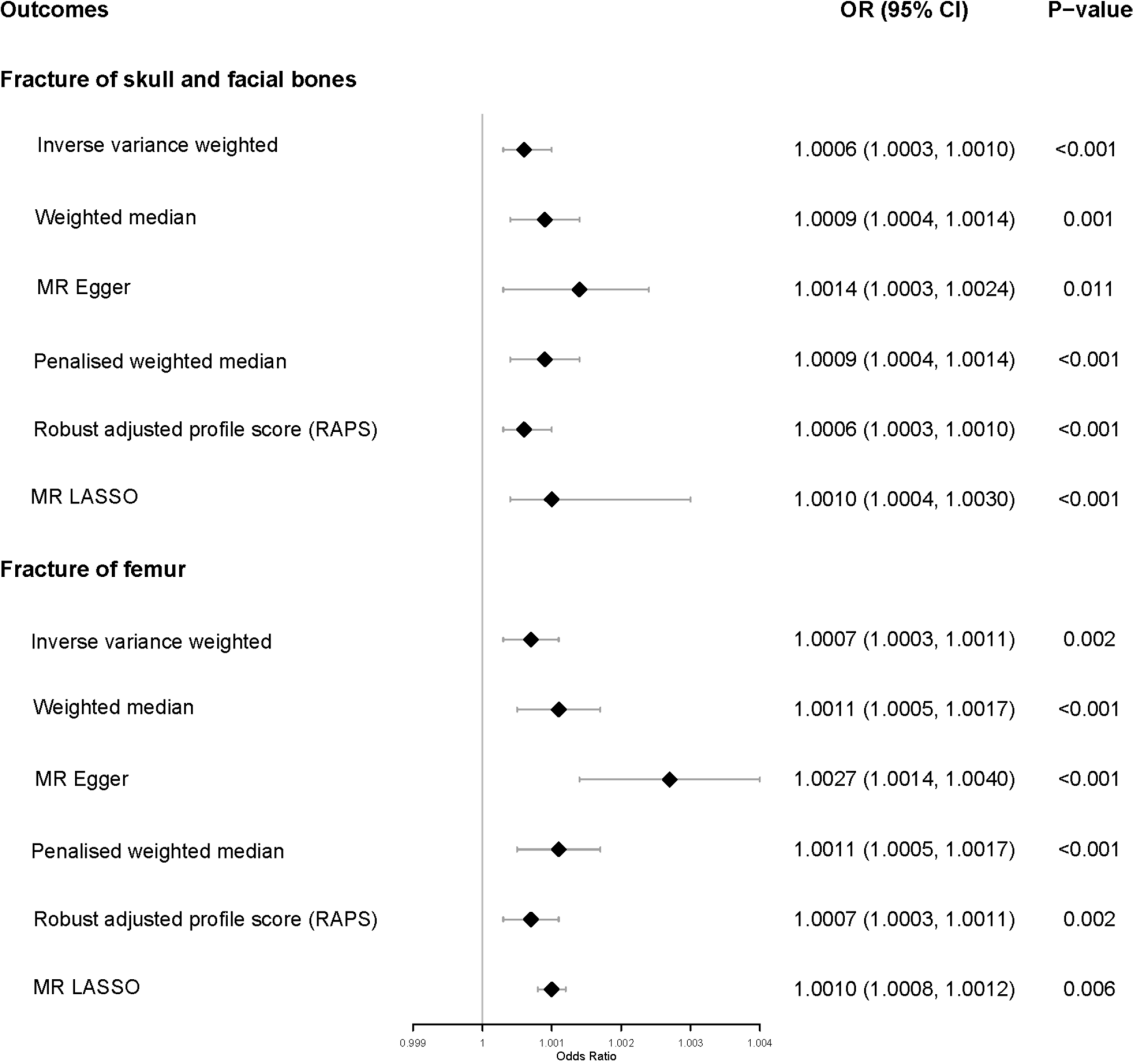
Forest plot of two-sample MR of the causal effect of schizophrenia on fracture

No causal effect of schizophrenia on mixed fracture was found (IVW OR (95% CI): 1.0029 (0.9845, 1.0217), *P* = 0.757); Table S2). We performed further analyses regarding the sites of the fracture and found that schizophrenia was a risk factor for fracture of the skull and facial bones (IVW OR (95% CI):1.0006(1.0003, 1.0010), *P* < 0.001), and femur (IVW OR (95% CI):1.0007(1.0003, 1.0011), *P* = 0.002) in all our implemented methods (Fig. 2). MR PRESSO detected no outliers (*P* = 0.362) and indicated no significant evidence of horizontal pleiotropy (*P* = 0.136). MR-Egger regression intercept which was unexpectedly close to zero suggested horizontal pleiotropy on fracture of femur (*P* < 0.001). However, the result of MR RAPS showed robust to horizontal pleiotropy (*P* = 0.002) and supported the causal effect. Heterogeneity tests revealed the lack of heterogeneity in fracture of skull and facial bones (IVW, Q = 173.749, *P* = 0.617) and femur (IVW, Q = 201.806, *P* = 0.138). Our results showed no causal effect of schizophrenia on fracture of the shoulder and upper arm or wrist and hand (IVW OR (95% CI): 0.9999 (0.9995–1.0003), *P* = 0.684; IVW OR (95% CI): 1.0002 (0.9998–1.0006), *P* = 0.247; Table S2). MR-Steiger tests indicated no reverse causality as P values were all less than 0.001. The results of the heterogeneity test, MR PRESSO, MR-Egger, MR-Steiger test and power were detailed in Supplementary Table S3.

### Two-sample MR for the causal effect of schizophrenia on BMD

**Fig. 3:**
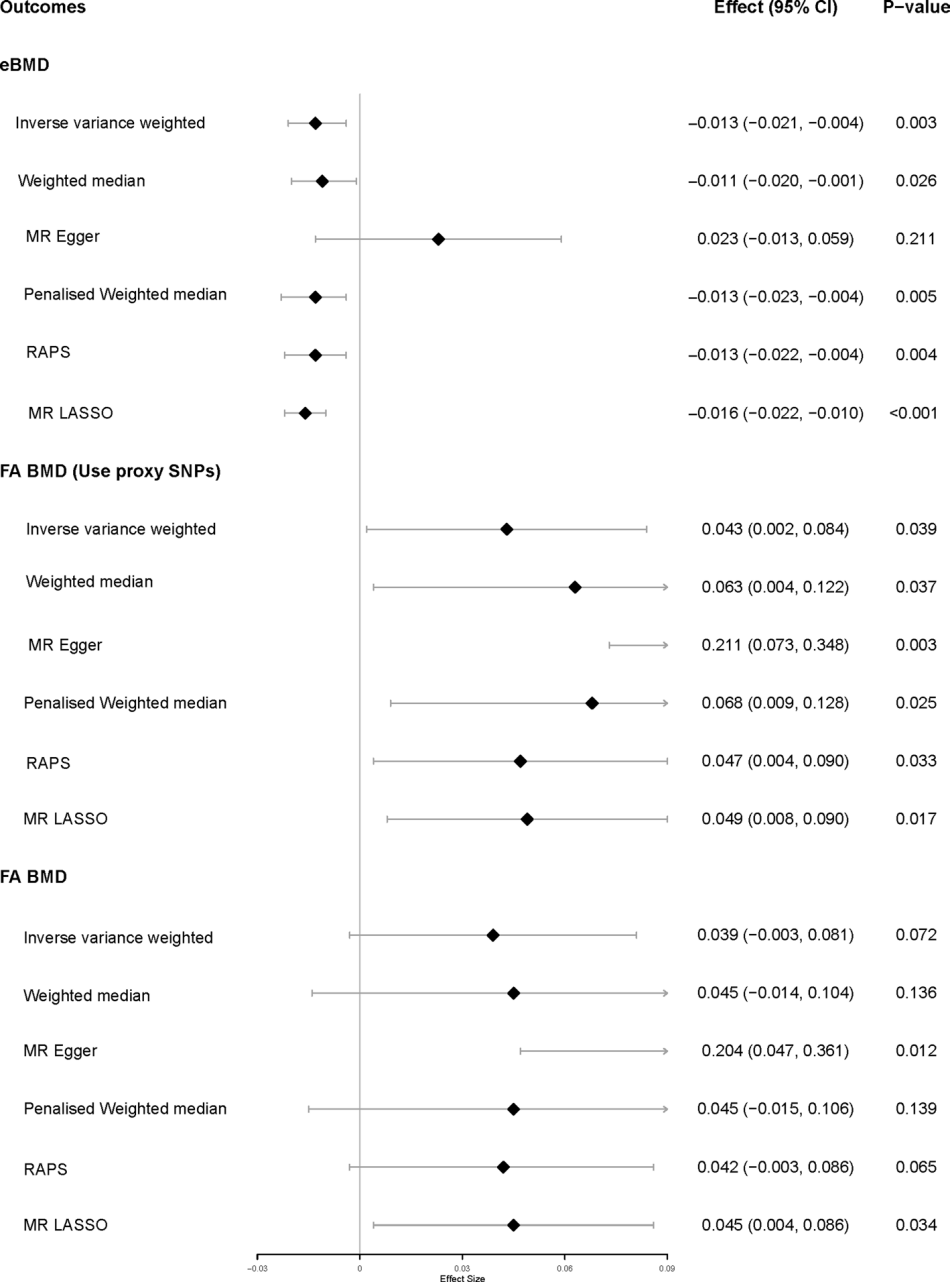
Forest plot of two-sample MR of the causal effects of schizophrenia on eBMD and FA BMD

MR analysis demonstrated that schizophrenia was negatively associated with the level of eBMD (IVW β (95%CI): -0.013 (-0.021, -0.004), *P* = 0.003) except MR-Egger (see Fig. 3). MR PRESSO and MR-Egger’s intercept detected evidence of pleiotropy (global test *P* < 0.001, intercept=-0.002, *P* = 0.048). In addition, heterogeneity was detected (based IVW: Q = 354.250, *P* < 0.001). However, the methods robust to heterogeneity and pleiotropy still showed a causal effect (P_Penalised WM_=0.026, P_MR LASSO_<0.001), with details in Supplementary Table S2. Only MR-Egger and MR LASSO demonstrated a casual relation between schizophrenia and FA BMD (P_MR−Egger_=0.012; P_MR LASSO_=0.035), while IVW and RAPS indicated a suggestive relationship (P_IVW_=0.072; P_RAPS_=0.065). We found that 5 SNPs were not extracted in FA BMD. Thus modifications were made. After using proxy SNPs, the causality of schizophrenia and FA BMD was identified in all methods (Fig. 3). However, no causal effect of schizophrenia on FN BMD or LS-BMD (IVW: P_FN BMD_=0.932; P_LS BMD_=0.935) was found (Table S4). No reverse causality was detected by means of the MR-Steiger test. The results of the heterogeneity test, MR PRESSO, MR-Egger, MR-Steiger test and power were detailed in Supplementary Table S3.

### Multivariable MR of schizophrenia with fracture and BMD after adjusting for BMI

To identify the potential sources of horizontal pleiotropy, we submitted all SNPs associated with schizophrenia (*n* = 189) to the PhenoScanner database, and 32 SNPs were identified to be associated with BMI, which was chosen for MVMR analysis. After adjusting for BMI, we still found a significant causal effect of schizophrenia on fracture of the skull and facial bones, fracture of the femur and eBMD (*P* = 0.009, *P* = 0.006, *P* = 0.035; Table S5).

## Discussion

Most of previous studies on schizophrenia increasing the risk of fracture were observational studies, whose results might be affected by behavioral factors such as vitamin D deficiency, physical activity, antipsychotics and diet [29–31]. To the best of our knowledge, the present study was the first MR study to systematically investigate the causality of genetic liability to schizophrenia with fracture and BMD in identical ancestry. Our results showed that schizophrenia was positively associated with the fracture of the skull and facial, and femur, whereas, inversely associated with eBMD. Prevention, early detection, and intervention are required.

A previous MR study found no causal relationship between schizophrenia and BMD; however, the lineage of a schizophrenia GWAS (Europe and Asia) and BMD GWAS (only Europe) was not uniform [32]. We only extracted European GWAS, to avoid the defect of unified ancestors, and identified that schizophrenia reduced the level of eBMD. The present result was also supported by a series of previous studies. For example, a cross-sectional study suggested a higher prevalence of osteoporosis and low bone mass in schizophrenia patients by measuring eBMD [3]. A meta-analysis in Western countries also showed that BMD level in patients with schizophrenia was significantly lower than that in age- and sex-matched controls [33]. Moreover, we found that schizophrenia was related to increased FA BMD. We believed that this result should be worth pondering because of the small sample size of FA BMD which might cause more susceptibility to environmental factors and the use of proxy SNP. Thus, our findings still needed to be further validated in larger samples and other populations.

Our study also found that schizophrenia had a genetic causal effect on the fracture of femur which was a position of the hip joint fracture. A recent 10-year cohort study suggested that the risk of hip fracture in schizophrenic patients was higher than that in the control group, while no difference was found in wrist fracture [5]. The evidence of schizophrenics prone to hip fractures compared to the general population also existed in a large population-cohort study [31]. Moreover, patients with comorbid schizophrenia had increased risks of unconventional discharge, adverse events and death after femoral fracture surgery, which might impose additional economic burdens on the healthcare system [34]. Currently, few studies on the fracture of skull and facial of schizophrenia were implemented, and we first discovered the causal effect of schizophrenia on the fracture of skull and facial. A cross-sectional study with a large sample showed that the proportion of primary nasal fractures in patients with mental illness was much higher and more severe [35]. Additionally, genetic variation such as apolipoprotein E, which was associated with the risk of Alzheimer’s disease, has shown a variable interaction with mild traumatic brain injury [36]. We speculated that there might be shared genetic variation between mental disorders that could help to explain the susceptibility to the fracture of skull.

It was worth discussing the reasons for the increased fracture and reduction of eBMD in schizophrenia patients. The possible mechanism underlying involved altered levels of inflammatory cytokines in schizophrenia to cause osteoclast formation and then increase the risk of fracture. Abnormal levels of inflammatory factors including C-reactive protein, transforming growth factor α(TGF- α), interleukin-6 (IL-6) and insulin-like growth factor (IGF-I) in schizophrenia were confirmed in the observational and MR studies [11, 37, 38]. Meanwhile, IGF-I was produced by osteoblasts and was involved in bone metabolism [39], TNF-α and IL-6 also partaken in bone resorption [40], ultimately leading to deficiency-related bone loss [41, 42]. With the IVs we selected, rs12833624, rs1615350, rs4947336, rs9258375, rs13195402, rs13195636, rs1264347, rs9461856, rs3795310, rs8192589 and rs3814883 were related to the lymphocytes, eosinophils and deficiency of IGA, which indirectly supported that inflammatory reaction might mediate schizophrenia and increase the risk of fracture.

In addition to the inflammatory process, a disturbed hypothalamic-pituitary-adrenocortical (HPA) axis might be another hypothesis for the risk of fracture in schizophrenia. Chronic psychological stress in schizophrenia affected the HPA axis, sympathetic nervous system, endocrine and immune factors, inhibited the secretion of gonadal hormone and growth hormone, and increased inflammatory cytokines, which might finally lead to bone loss by inhibiting bone formation and stimulating bone absorption [43]. Besides, the endocrine cannabinoid system played an important role in the development of schizophrenia, Cannabinoid receptors type 1 has been widely found to be involved in regulating the HPA axis in the meso solute pathway [44]. HPA axis activity was increased in patients with mental disorders, which was particularly common at the time of onset [45]. Hence, we speculated that schizophrenic patients might, directly and indirectly, affect bone cells through HPA axis, thus increasing fracture risk and reducing BMD.

The advantage of this study derived from the manipulation of MR, to minimize the confounding factors and reverse causal effects in observational studies, and a series of sensitivity analysis methods were considered to obtain robust results, weakening the limitations of heterogeneity and horizontal pleiotropy. The cases of fractures in various parts were diagnosed according to the international standard ICD-10 rather than self-reported. All individuals in the study unified into European populations to avoid ethnic mixing. Otherwise, it might be inaccurate to extend our conclusions to other populations. Although we had strenuously collected the largest sample of open fracture GWAS in all parts, the number of cases was still limited to accurately determine the level of its causal effect. Thus, these findings still needed to be confirmed. In addition, our findings were identified on heel eBMD which did not represent BMD, despite ultrasound eBMD was increasingly recognized as a valid alternative to DXA [46] and successfully wielded in previous studies [16, 18, 47]. Though previous observational studies believed that schizophrenia would increase the risk of fracture and reduce BMD [5, 29, 48], our results still needed to be confirmed in future studies. Finally, only BMI was considered in MVMR as it was mostly correlated with the selected IVs, but the effects of other confounding factors such as age, and vitamin D were not included, which was worth further investigated.

## Conclusion

To sum up, the current finding confirmed that schizophrenia was causally associated with the fractures of skull and facial bone, fracture of femur as well as the eBMD. The causal effects still existed after adjusting for BMI. This study might further remind clinicians to keep elevated fracture risk in mind when managing schizophrenic patients, which would help formulate new strategies to improve the lifestyle and bone health of patients.

### Supplementary Information

**Additional file 1: Table S1.** Instrumental variables used for Mendelian randomization. **Table S2.** Mendelian randomization results for causal effect of schizophrenia on fracture. IVW, inverse-variance weighted; WM, Weighted median. **Table S3.** Results of heterogeneity test, sensitivity analysis and power for Mendelian. **Table S4.** Mendelian randomization results for causal effect of schizophrenia on FN and LS BMD IVW, inverse-variance weighted; WM, Weighted median. **Table S5.** Results of MVMR analyses of the causal effect of schizophrenia on fracture and BMD adjusting for BMI.

## Data Availability

Summary data on schizophrenia is available on Psychiatric Genomics Consortium (PGC: https://figshare.com/articles/dataset/scz2022/19426775). Mixed fracture, eBMD, FA, FN and LS BMD GWAS could be obtained from GEFOS) consortium (http://www.gefos.org). Site-specific fracture data are downloaded from UK Biobank (http://www.nealelab.is/uk-biobank). The GWAS data of BMI is available on (GIANT) consortium (https://portals.broadinstitute.org/collaboration/giant/index.php/GIANT_consortium_data_files).

## Abbreviations

BMD: Bone mineral density
MR: Mendelian randomization
GWAS: Genome-wide association study
FA: Forearm
FN: Femoral neck
LS: Lumbar spine
eBMD: estimated BMD
MVMR: Multivariable Mendelian randomization
BMI: Body mass index
IVW: Inverse variance weighting
OR: Odds ratio
CI: Confidence interval
