# Supplementary material for: The causal effect of schizophrenia on fractures and bone mineral density: a comprehensive two-sample Mendelian randomization study of European ancestry
Source: BMC Psychiatry. 2023 Sep 25;23:692. doi: 10.1186/s12888-023-05196-8 (PMC10518911; doi:10.1186/s12888-023-05196-8)
Supplement: Supplementary file 1 — Additional file 1: Table S1. Instrumental variables used for Mendelian randomization. Table S2. Mendelian randomization results for causal effect of schizophrenia on fracture. IVW, inverse-variance weighted; WM, Weighted median. Table S3. Results of heterogeneity test, sensitivity analysis and power for Mendelian. Table S4. Mendelian randomization results for causal effect of schizophrenia on FN and LS BMD IVW, inverse-variance weighted; WM, Weighted median. Table S5. Results of MVMR analyses of the causal effect of schizophrenia on fracture and BMD adjusting for BMI. [file 12888_2023_5196_MOESM1_ESM.docx]

Table S1. Instrumental variables used for Mendelian randomization

| SNP | effect_allele | other_allele | Beta | Se | P-val | Sample size | Eaf | F_statistics | R^2^ |
| --- | --- | --- | --- | --- | --- | --- | --- | --- | --- |
| rs6715366 | G | A | -0.054 | 0.010 | 2.49E-08 | 130644 | 0.729 | 31.10 | 0.0012 |
| rs113113059 | T | C | 0.058 | 0.011 | 4.89E-08 | 130644 | 0.791 | 29.68 | 0.0011 |
| rs10873538 | T | G | -0.067 | 0.009 | 3.01E-13 | 130644 | 0.661 | 53.41 | 0.0020 |
| rs2532240 | C | T | 0.061 | 0.009 | 2.58E-11 | 130644 | 0.602 | 44.65 | 0.0018 |
| rs1892346 | T | A | -0.048 | 0.009 | 3.56E-08 | 130644 | 0.433 | 30.25 | 0.0012 |
| rs7515363 | C | T | 0.054 | 0.009 | 1.84E-09 | 130644 | 0.384 | 36.14 | 0.0014 |
| rs11687313 | G | A | -0.095 | 0.013 | 2.84E-14 | 130644 | 0.864 | 57.44 | 0.0021 |
| rs56335113 | A | G | 0.065 | 0.009 | 6.02E-12 | 130644 | 0.308 | 47.38 | 0.0018 |
| rs11263861 | G | A | -0.052 | 0.009 | 2.02E-08 | 130644 | 0.323 | 31.39 | 0.0012 |
| rs1915019 | A | G | 0.057 | 0.010 | 6.57E-09 | 130644 | 0.256 | 33.95 | 0.0012 |
| rs308697 | C | A | 0.050 | 0.009 | 8.83E-09 | 130644 | 0.568 | 33.17 | 0.0012 |
| rs12833624 | C | T | -0.050 | 0.009 | 2.77E-08 | 130644 | 0.653 | 31.11 | 0.0011 |
| rs1615350 | C | T | 0.074 | 0.010 | 4.92E-14 | 130644 | 0.263 | 56.41 | 0.0021 |
| rs6001259 | C | T | -0.191 | 0.035 | 3.70E-08 | 126538 | 0.983 | 30.28 | 0.0012 |
| rs167924 | A | G | -0.050 | 0.009 | 2.34E-08 | 130644 | 0.365 | 31.11 | 0.0012 |
| rs72943392 | G | C | -0.053 | 0.010 | 2.39E-08 | 130644 | 0.709 | 31.05 | 0.0012 |
| rs9876421 | C | T | -0.063 | 0.009 | 9.19E-12 | 130644 | 0.652 | 46.16 | 0.0018 |
| rs6549963 | T | C | 0.048 | 0.009 | 4.31E-08 | 130644 | 0.589 | 30.13 | 0.0011 |
| rs6538539 | G | T | 0.057 | 0.009 | 4.43E-11 | 130644 | 0.455 | 43.62 | 0.0016 |
| rs10777956 | A | G | -0.050 | 0.009 | 1.75E-08 | 130644 | 0.376 | 31.56 | 0.0012 |
| rs7575796 | A | G | 0.096 | 0.017 | 2.07E-08 | 130644 | 0.910 | 31.35 | 0.0015 |
| rs10086619 | A | G | -0.072 | 0.012 | 4.97E-10 | 130644 | 0.836 | 38.75 | 0.0014 |
| rs4702 | G | A | 0.084 | 0.009 | 2.79E-21 | 130644 | 0.449 | 89.73 | 0.0035 |
| rs11210892 | G | A | 0.064 | 0.009 | 2.68E-12 | 130644 | 0.337 | 48.69 | 0.0018 |
| rs11136325 | G | A | 0.054 | 0.009 | 3.05E-09 | 130644 | 0.427 | 34.95 | 0.0014 |
| rs4129585 | A | C | 0.075 | 0.009 | 5.11E-18 | 130644 | 0.445 | 74.31 | 0.0028 |
| rs12303743 | G | C | -0.087 | 0.015 | 1.59E-09 | 130644 | 0.903 | 36.41 | 0.0013 |
| rs217336 | C | A | 0.050 | 0.009 | 8.05E-09 | 130644 | 0.573 | 33.43 | 0.0012 |
| rs3824451 | T | C | -0.066 | 0.012 | 2.54E-08 | 130644 | 0.843 | 30.90 | 0.0011 |
| rs2381411 | T | C | -0.050 | 0.009 | 1.25E-08 | 130644 | 0.593 | 32.80 | 0.0012 |
| rs39967 | T | C | -0.062 | 0.011 | 4.38E-08 | 130644 | 0.175 | 29.96 | 0.0011 |
| rs77502336 | G | C | -0.053 | 0.009 | 1.25E-08 | 130644 | 0.680 | 32.23 | 0.0012 |
| rs12293670 | A | G | 0.070 | 0.009 | 1.56E-14 | 130644 | 0.667 | 58.72 | 0.0022 |
| rs11740474 | A | T | -0.054 | 0.009 | 1.13E-09 | 130644 | 0.581 | 37.23 | 0.0014 |
| rs72802868 | G | T | 0.069 | 0.010 | 4.55E-13 | 130644 | 0.713 | 51.96 | 0.0020 |
| rs12652777 | T | C | 0.049 | 0.009 | 1.52E-08 | 130644 | 0.482 | 32.20 | 0.0012 |
| rs149165 | T | G | 0.048 | 0.009 | 3.01E-08 | 130644 | 0.560 | 30.69 | 0.0011 |
| rs12771371 | G | A | 0.052 | 0.009 | 1.94E-08 | 130644 | 0.692 | 31.75 | 0.0012 |
| rs7830315 | T | C | -0.048 | 0.009 | 3.08E-08 | 130644 | 0.485 | 30.90 | 0.0011 |
| rs6984242 | G | A | 0.055 | 0.009 | 3.86E-10 | 130644 | 0.405 | 39.53 | 0.0014 |
| rs11941714 | G | A | 0.052 | 0.009 | 3.07E-08 | 130644 | 0.668 | 30.78 | 0.0012 |
| rs58120505 | T | C | 0.090 | 0.009 | 2.24E-24 | 130644 | 0.590 | 103.68 | 0.0039 |
| rs17731 | G | A | -0.052 | 0.009 | 4.37E-09 | 130644 | 0.629 | 34.66 | 0.0013 |
| rs4766428 | C | T | -0.075 | 0.009 | 3.93E-17 | 130644 | 0.551 | 71.02 | 0.0028 |
| rs7251 | C | G | 0.064 | 0.009 | 8.29E-12 | 130644 | 0.676 | 46.50 | 0.0018 |
| rs2999392 | C | T | -0.052 | 0.009 | 3.05E-08 | 130644 | 0.305 | 30.37 | 0.0011 |
| rs2333321 | A | G | 0.071 | 0.011 | 1.25E-11 | 130644 | 0.212 | 45.99 | 0.0017 |
| rs61405217 | C | T | 0.050 | 0.009 | 7.03E-09 | 130644 | 0.464 | 33.67 | 0.0012 |
| rs1427633 | G | C | 0.048 | 0.009 | 4.10E-08 | 130644 | 0.412 | 30.13 | 0.0011 |
| rs3791710 | T | C | 0.060 | 0.011 | 3.02E-08 | 130644 | 0.800 | 30.87 | 0.0012 |
| rs1384292 | G | C | -0.049 | 0.009 | 3.05E-08 | 130644 | 0.545 | 30.75 | 0.0012 |
| rs6943762 | T | C | 0.105 | 0.013 | 1.57E-15 | 130644 | 0.875 | 63.39 | 0.0024 |
| rs13233308 | C | T | 0.049 | 0.009 | 1.75E-08 | 130644 | 0.520 | 32.07 | 0.0012 |
| rs2252074 | T | G | -0.069 | 0.009 | 6.19E-15 | 130644 | 0.599 | 60.60 | 0.0023 |
| rs16851048 | T | C | -0.074 | 0.011 | 4.15E-12 | 130644 | 0.800 | 48.47 | 0.0018 |
| rs4636654 | G | A | 0.048 | 0.009 | 4.89E-08 | 130644 | 0.602 | 29.46 | 0.0011 |
| rs11027839 | A | C | -0.052 | 0.009 | 2.40E-09 | 130644 | 0.495 | 35.87 | 0.0013 |
| rs778371 | A | G | -0.081 | 0.010 | 1.50E-17 | 130644 | 0.708 | 71.99 | 0.0027 |
| rs4575535 | A | G | -0.056 | 0.010 | 5.77E-09 | 130644 | 0.290 | 33.78 | 0.0013 |
| rs2456020 | C | T | 0.082 | 0.010 | 1.13E-15 | 130644 | 0.765 | 64.00 | 0.0024 |
| rs10117 | G | A | 0.055 | 0.009 | 4.66E-10 | 130644 | 0.606 | 39.06 | 0.0014 |
| rs9687282 | T | G | -0.053 | 0.009 | 7.33E-09 | 130644 | 0.659 | 33.41 | 0.0012 |
| rs4947336 | T | A | 0.208 | 0.016 | 8.84E-38 | 130644 | 0.917 | 165.17 | 0.0066 |
| rs9258375 | A | G | 0.188 | 0.016 | 1.80E-32 | 130644 | 0.906 | 141.28 | 0.0060 |
| rs13195402 | G | T | 0.210 | 0.017 | 5.74E-36 | 130644 | 0.924 | 156.70 | 0.0062 |
| rs62392365 | A | T | 0.172 | 0.020 | 2.49E-17 | 127074 | 0.948 | 71.45 | 0.0029 |
| rs13195636 | A | C | 0.211 | 0.016 | 6.55E-40 | 130644 | 0.916 | 175.28 | 0.0068 |
| rs35531336 | A | G | 0.074 | 0.013 | 2.22E-08 | 130644 | 0.867 | 31.34 | 0.0013 |
| rs728055 | T | A | 0.067 | 0.009 | 8.85E-14 | 130644 | 0.649 | 56.08 | 0.0021 |
| rs7801375 | A | G | -0.073 | 0.012 | 7.56E-10 | 130644 | 0.157 | 37.84 | 0.0014 |
| rs1264347 | C | T | 0.162 | 0.015 | 1.03E-28 | 130644 | 0.890 | 123.12 | 0.0052 |
| rs9461856 | G | A | -0.062 | 0.009 | 5.71E-13 | 130644 | 0.484 | 52.14 | 0.0019 |
| rs8192589 | G | T | 0.152 | 0.014 | 2.43E-29 | 130644 | 0.875 | 125.73 | 0.0051 |
| rs11693094 | C | T | 0.054 | 0.009 | 4.29E-10 | 130644 | 0.546 | 39.10 | 0.0015 |
| rs12129573 | C | A | -0.078 | 0.009 | 2.28E-18 | 130644 | 0.627 | 76.41 | 0.0028 |
| rs215412 | G | A | -0.058 | 0.009 | 2.69E-10 | 130644 | 0.668 | 40.21 | 0.0015 |
| rs7647398 | C | T | 0.077 | 0.011 | 1.07E-12 | 130644 | 0.804 | 50.55 | 0.0019 |
| rs1430894 | C | T | -0.053 | 0.009 | 6.15E-10 | 130644 | 0.513 | 38.40 | 0.0014 |
| rs12489270 | T | C | -0.058 | 0.009 | 7.47E-11 | 130644 | 0.620 | 42.33 | 0.0016 |
| rs5995756 | T | C | 0.057 | 0.009 | 3.18E-11 | 130644 | 0.446 | 44.55 | 0.0016 |
| rs133047 | T | C | 0.079 | 0.014 | 3.18E-08 | 130644 | 0.105 | 30.60 | 0.0012 |
| rs5751191 | T | C | -0.066 | 0.009 | 3.00E-14 | 130644 | 0.493 | 58.18 | 0.0022 |
| rs699318 | T | C | 0.067 | 0.009 | 2.27E-13 | 130644 | 0.328 | 53.35 | 0.0020 |
| rs1451488 | A | G | -0.071 | 0.009 | 4.47E-16 | 130644 | 0.440 | 66.40 | 0.0025 |
| rs3770754 | C | G | 0.053 | 0.009 | 5.35E-09 | 130644 | 0.637 | 33.79 | 0.0013 |
| rs4812325 | G | A | -0.072 | 0.009 | 8.96E-16 | 130644 | 0.378 | 65.27 | 0.0024 |
| rs60135207 | G | T | 0.050 | 0.009 | 1.53E-08 | 130644 | 0.589 | 31.77 | 0.0012 |
| rs16825349 | A | G | -0.069 | 0.011 | 7.72E-10 | 130644 | 0.820 | 37.84 | 0.0014 |
| rs13016542 | T | C | 0.088 | 0.013 | 8.28E-12 | 130644 | 0.869 | 46.86 | 0.0018 |
| rs500102 | T | C | 0.052 | 0.009 | 4.87E-09 | 130644 | 0.411 | 34.52 | 0.0013 |
| rs2078266 | A | G | 0.070 | 0.013 | 2.94E-08 | 130644 | 0.174 | 30.51 | 0.0014 |
| rs7900775 | T | C | 0.049 | 0.009 | 3.82E-08 | 130644 | 0.364 | 30.31 | 0.0011 |
| rs11191580 | T | C | 0.132 | 0.016 | 1.77E-17 | 130644 | 0.914 | 72.20 | 0.0027 |
| rs9454727 | A | G | 0.054 | 0.010 | 3.35E-08 | 130644 | 0.731 | 30.82 | 0.0012 |
| rs2815731 | C | A | 0.060 | 0.009 | 4.39E-11 | 130644 | 0.649 | 43.48 | 0.0016 |
| rs10876446 | G | C | -0.054 | 0.009 | 1.03E-08 | 130644 | 0.682 | 33.00 | 0.0013 |
| rs61937595 | C | T | 0.130 | 0.016 | 1.15E-15 | 130644 | 0.912 | 64.49 | 0.0027 |
| rs73292401 | T | A | -0.068 | 0.011 | 5.48E-10 | 130644 | 0.804 | 38.47 | 0.0014 |
| rs57433322 | C | G | 0.083 | 0.014 | 1.99E-09 | 130644 | 0.881 | 35.74 | 0.0014 |
| rs8055219 | G | A | -0.067 | 0.010 | 5.69E-11 | 130644 | 0.766 | 43.36 | 0.0016 |
| rs187557 | C | T | 0.067 | 0.012 | 2.03E-08 | 130644 | 0.156 | 31.41 | 0.0012 |
| rs1901512 | T | C | 0.058 | 0.009 | 5.72E-10 | 130644 | 0.308 | 38.60 | 0.0015 |
| rs10861176 | G | A | -0.056 | 0.010 | 1.59E-08 | 130644 | 0.261 | 32.08 | 0.0012 |
| rs10860960 | C | T | 0.052 | 0.009 | 6.26E-09 | 130644 | 0.630 | 34.01 | 0.0013 |
| rs2455415 | C | T | -0.049 | 0.009 | 1.69E-08 | 130644 | 0.583 | 31.63 | 0.0012 |
| rs1604060 | A | G | -0.077 | 0.014 | 3.24E-08 | 130644 | 0.110 | 30.41 | 0.0012 |
| rs10035564 | A | G | -0.067 | 0.009 | 4.38E-13 | 130644 | 0.661 | 52.72 | 0.0020 |
| rs1540840 | G | C | 0.056 | 0.009 | 2.21E-09 | 130644 | 0.532 | 35.87 | 0.0015 |
| rs17194490 | G | T | -0.078 | 0.012 | 1.80E-11 | 130644 | 0.834 | 45.45 | 0.0017 |
| rs61857878 | A | T | 0.060 | 0.010 | 4.44E-09 | 130644 | 0.752 | 34.71 | 0.0013 |
| rs2514218 | C | T | 0.070 | 0.009 | 1.35E-14 | 130644 | 0.661 | 58.72 | 0.0022 |
| rs1881046 | G | T | 0.051 | 0.009 | 3.39E-08 | 130644 | 0.664 | 30.37 | 0.0011 |
| rs79210963 | T | C | -0.086 | 0.014 | 4.14E-10 | 130644 | 0.889 | 39.04 | 0.0014 |
| rs12285419 | C | A | -0.085 | 0.011 | 1.05E-14 | 130644 | 0.807 | 59.58 | 0.0022 |
| rs3729986 | C | T | -0.088 | 0.015 | 2.18E-09 | 130644 | 0.902 | 35.92 | 0.0014 |
| rs634940 | G | T | -0.066 | 0.010 | 1.78E-11 | 130644 | 0.744 | 44.98 | 0.0017 |
| rs6925964 | A | T | 0.098 | 0.018 | 3.11E-08 | 130644 | 0.935 | 30.75 | 0.0012 |
| rs9304548 | C | A | 0.057 | 0.010 | 1.59E-08 | 130644 | 0.256 | 32.15 | 0.0012 |
| rs2710323 | T | C | 0.078 | 0.009 | 1.23E-19 | 130644 | 0.519 | 83.12 | 0.0031 |
| rs7634476 | A | G | -0.058 | 0.009 | 5.46E-11 | 130644 | 0.407 | 43.00 | 0.0016 |
| rs4779050 | T | G | 0.058 | 0.009 | 7.27E-11 | 130644 | 0.373 | 42.46 | 0.0016 |
| rs11638554 | T | G | 0.065 | 0.010 | 7.58E-12 | 130644 | 0.707 | 46.96 | 0.0018 |
| rs6673880 | A | G | -0.062 | 0.009 | 7.20E-12 | 130644 | 0.500 | 46.87 | 0.0019 |
| rs3795310 | C | T | 0.051 | 0.009 | 5.75E-09 | 130644 | 0.539 | 34.36 | 0.0013 |
| rs708228 | C | T | -0.053 | 0.009 | 6.56E-09 | 130644 | 0.667 | 33.67 | 0.0012 |
| rs246024 | C | T | 0.048 | 0.009 | 3.61E-08 | 130644 | 0.493 | 30.51 | 0.0011 |
| rs11993663 | C | A | -0.050 | 0.009 | 3.32E-08 | 130644 | 0.667 | 30.67 | 0.0011 |
| rs4921741 | A | G | -0.056 | 0.010 | 1.21E-08 | 130644 | 0.735 | 32.65 | 0.0012 |
| rs72974238 | C | A | 0.053 | 0.009 | 8.74E-09 | 130644 | 0.673 | 32.94 | 0.0012 |
| rs35351411 | A | C | -0.064 | 0.009 | 2.21E-13 | 130644 | 0.449 | 53.28 | 0.0020 |
| rs3814883 | C | T | 0.067 | 0.009 | 1.58E-14 | 130644 | 0.539 | 59.48 | 0.0022 |
| rs2332700 | C | G | 0.075 | 0.010 | 3.88E-14 | 130644 | 0.251 | 57.54 | 0.0021 |
| rs1000237 | T | A | -0.073 | 0.009 | 2.80E-16 | 130644 | 0.638 | 67.66 | 0.0025 |
| rs72986630 | C | T | -0.112 | 0.018 | 3.59E-10 | 130644 | 0.930 | 39.36 | 0.0017 |
| rs7191183 | T | C | -0.058 | 0.009 | 3.32E-10 | 130644 | 0.686 | 39.44 | 0.0015 |
| rs12138231 | T | A | -0.067 | 0.012 | 7.99E-09 | 130644 | 0.178 | 33.36 | 0.0013 |
| rs13107325 | C | T | -0.159 | 0.017 | 2.90E-21 | 129529 | 0.927 | 89.24 | 0.0034 |
| rs117799466 | G | C | -0.062 | 0.010 | 1.28E-10 | 130644 | 0.652 | 41.71 | 0.0017 |
| rs11807834 | G | A | -0.055 | 0.010 | 2.98E-08 | 130644 | 0.733 | 30.65 | 0.0012 |
| rs11587347 | C | G | -0.104 | 0.015 | 1.53E-12 | 130644 | 0.902 | 49.95 | 0.0019 |
| rs145071536 | T | C | -0.085 | 0.012 | 1.62E-12 | 130644 | 0.804 | 50.29 | 0.0023 |
| rs6482437 | A | C | -0.099 | 0.014 | 3.33E-12 | 130644 | 0.107 | 48.51 | 0.0019 |
| rs16867571 | A | G | 0.066 | 0.010 | 2.68E-10 | 130644 | 0.770 | 39.91 | 0.0015 |
| rs7783665 | G | A | 0.051 | 0.009 | 6.13E-09 | 130644 | 0.590 | 34.09 | 0.0012 |
| rs7803571 | C | T | 0.064 | 0.009 | 1.27E-12 | 130644 | 0.627 | 49.94 | 0.0019 |
| rs1914399 | C | G | 0.049 | 0.009 | 1.40E-08 | 130644 | 0.483 | 31.86 | 0.0012 |
| rs4700418 | C | G | -0.070 | 0.009 | 5.37E-16 | 130644 | 0.500 | 65.10 | 0.0025 |
| rs73229090 | C | A | 0.103 | 0.014 | 4.34E-13 | 130644 | 0.892 | 52.21 | 0.0020 |
| rs113264400 | T | C | -0.112 | 0.020 | 2.87E-08 | 130644 | 0.950 | 30.90 | 0.0012 |
| rs13011472 | C | G | -0.070 | 0.009 | 4.28E-16 | 130644 | 0.509 | 65.48 | 0.0025 |
| rs4632195 | C | T | -0.047 | 0.009 | 4.59E-08 | 130644 | 0.476 | 30.12 | 0.0011 |
| rs9636107 | A | G | -0.070 | 0.009 | 5.12E-16 | 130644 | 0.520 | 66.06 | 0.0024 |
| rs17571951 | T | C | -0.064 | 0.011 | 9.97E-10 | 130644 | 0.782 | 37.38 | 0.0014 |
| rs12883788 | C | T | -0.061 | 0.009 | 1.86E-12 | 130644 | 0.536 | 49.65 | 0.0019 |
| rs2053079 | A | G | -0.060 | 0.010 | 3.01E-09 | 130644 | 0.759 | 35.17 | 0.0013 |
| rs505061 | C | A | -0.053 | 0.009 | 5.80E-10 | 130644 | 0.505 | 38.69 | 0.0014 |
| rs12877581 | G | C | -0.060 | 0.010 | 1.80E-09 | 130644 | 0.720 | 36.24 | 0.0014 |
| rs9318627 | A | C | 0.061 | 0.009 | 4.35E-12 | 130644 | 0.605 | 48.36 | 0.0018 |
| rs6546857 | A | G | -0.060 | 0.010 | 2.74E-09 | 130644 | 0.764 | 35.06 | 0.0013 |
| rs17016552 | C | G | 0.052 | 0.009 | 1.20E-08 | 130644 | 0.657 | 32.28 | 0.0012 |
| rs1198588 | A | T | -0.103 | 0.011 | 1.73E-21 | 130644 | 0.205 | 90.25 | 0.0034 |
| rs11165867 | C | T | -0.074 | 0.012 | 1.30E-10 | 130644 | 0.836 | 41.03 | 0.0015 |
| rs56205728 | G | A | -0.063 | 0.010 | 1.01E-10 | 130644 | 0.708 | 42.19 | 0.0016 |
| rs2255663 | C | T | 0.058 | 0.010 | 8.40E-10 | 130644 | 0.707 | 37.54 | 0.0014 |
| rs62183855 | A | C | 0.066 | 0.011 | 2.66E-09 | 130644 | 0.810 | 35.46 | 0.0013 |
| rs331395 | C | G | -0.061 | 0.010 | 5.55E-09 | 130644 | 0.784 | 34.29 | 0.0013 |
| rs4672366 | A | T | 0.054 | 0.010 | 2.80E-08 | 130644 | 0.706 | 30.82 | 0.0012 |
| rs10108980 | C | T | -0.063 | 0.011 | 2.73E-09 | 130644 | 0.790 | 35.10 | 0.0013 |
| rs11664298 | G | A | -0.077 | 0.011 | 8.94E-13 | 130644 | 0.802 | 51.36 | 0.0019 |
| rs76838079 | C | T | -0.078 | 0.014 | 1.53E-08 | 130644 | 0.852 | 31.95 | 0.0015 |
| rs11603151 | G | A | -0.053 | 0.009 | 1.10E-08 | 130644 | 0.680 | 32.57 | 0.0012 |
| rs10894308 | G | A | 0.054 | 0.009 | 8.18E-10 | 130644 | 0.540 | 37.65 | 0.0014 |
| rs11223774 | A | G | 0.052 | 0.009 | 2.74E-08 | 130644 | 0.297 | 31.19 | 0.0012 |
| rs4936215 | A | G | 0.082 | 0.011 | 1.87E-14 | 130644 | 0.777 | 58.30 | 0.0023 |
| rs3016386 | G | A | 0.050 | 0.009 | 6.24E-09 | 130644 | 0.513 | 33.43 | 0.0013 |
| rs79445414 | T | C | -0.123 | 0.022 | 2.80E-08 | 130644 | 0.958 | 30.90 | 0.0012 |
| rs7001340 | T | C | 0.058 | 0.011 | 3.17E-08 | 130644 | 0.786 | 30.45 | 0.0011 |
| rs61786047 | G | A | 0.078 | 0.014 | 8.34E-09 | 130644 | 0.881 | 33.21 | 0.0013 |
| rs6520064 | A | G | -0.058 | 0.011 | 3.58E-08 | 130644 | 0.792 | 30.46 | 0.0011 |
| rs713692 | G | A | -0.057 | 0.010 | 2.67E-09 | 130263 | 0.303 | 35.50 | 0.0014 |
| rs6798742 | A | G | -0.061 | 0.009 | 4.57E-11 | 130644 | 0.688 | 43.16 | 0.0016 |
| rs741896 | C | G | -0.054 | 0.009 | 2.17E-09 | 130644 | 0.654 | 35.74 | 0.0013 |
| rs498591 | A | T | -0.072 | 0.012 | 2.11E-09 | 130644 | 0.851 | 35.90 | 0.0013 |
| rs2238057 | T | G | -0.084 | 0.009 | 8.50E-22 | 130644 | 0.581 | 92.12 | 0.0034 |
| rs12712510 | T | C | 0.057 | 0.009 | 5.14E-11 | 130644 | 0.479 | 43.53 | 0.0016 |
| rs2909457 | G | A | 0.049 | 0.009 | 1.48E-08 | 130644 | 0.442 | 31.72 | 0.0012 |
| rs35734242 | T | C | -0.051 | 0.009 | 1.37E-08 | 130644 | 0.569 | 32.46 | 0.0013 |
| rs6125656 | G | A | -0.064 | 0.011 | 6.29E-09 | 130644 | 0.815 | 33.76 | 0.0013 |
| rs926288 | A | G | -0.061 | 0.011 | 2.50E-08 | 130644 | 0.197 | 31.06 | 0.0012 |

Table S2. Mendelian randomization results for causal effect of schizophrenia on fracture

| Outcomes | Method | nSNP | OR (95% CI) | P |
| --- | --- | --- | --- | --- |
| Mixed fracture | IVW | 180 | 1.0029 (0.9845-1.0217) | 0.757 |
|  | WM | 180 | 1.0038 (0.9792-1.0290) | 0.766 |
|  | MR Egger | 180 | 1.0086 (0.9534-1.0671) | 0.765 |
|  | Penalised WM | 180 | 1.0062 (0.9812-1.0319) | 0.628 |
|  | RAPS | 180 | 1.0049 (0.9855-1.0246) | 0.623 |
|  | MR LASSO | 165 | 1.0060 (0.9884-1.0239) | 0.491 |
| Fracture of shoulder and upper arm | IVW | 182 | 0.9999 (0.9995-1.0003) | 0.684 |
|  | WM | 182 | 0.9997 (0.9991-1.0004) | 0.422 |
|  | MR Egger | 182 | 0.9985 (0.9972-0.9997) | 0.014 |
|  | Penalised WM | 182 | 0.9997 (0.9991-1.0004) | 0.407 |
|  | RAPS | 182 | 0.9999 (0.9995-1.0003) | 0.574 |
|  | MR LASSO | 182 | 1.0001 (0.9999-1.0003) | 0.684 |
| Fracture of wrist and hand | IVW | 182 | 1.0002 (0.9998-1.0006) | 0.247 |
|  | WM | 182 | 1.0007 (1.0001-1.0013) | 0.028 |
|  | MR Egger | 182 | 1.0014 (1.0002-1.0026) | 0.025 |
|  | Penalised WM | 182 | 1.0008 (1.0002-1.0014) | 0.015 |
|  | RAPS | 182 | 1.0004 (1.0000-1.0008) | 0.076 |
|  | MR LASSO | 179 | 1.0001 (0.9999-1.0013) | 0.094 |

IVW, inverse-variance weighted; WM, Weighted median.

Table S3. Results of heterogeneity test, sensitivity analysis and power for Mendelian randomization

| Outcomes | Heterogeneity (IVW) | | MR PRESSO | | MR Egger | | |  | MR-Steiger test | | |  | | |
| --- | --- | --- | --- | --- | --- | --- | --- | --- | --- | --- | --- | --- | --- | --- |
|  | Cochran Q statistic | P | P (Global Test) | intercept | | P | R2_  exposure | | | R2_  outcomes | P | | Power |  |
| Fracture | 244.762 | 0.0008 | <0.001 | 0.001 | | 0.834 | 0.0637 | | | 0.0006 | <0.001 | | 2.7% |  |
| Fracture of skull and face | 173.749 | 0.617 | 0.362 | 0.001 | | 0.140 | 0.0638 | | | 0.0005 | <0.001 | | 2.5% |  |
| Fracture of shoulder and upper arm | 176.673 | 0.577 | 0.575 | 0.001 | | 0.013 | 0.0643 | | | 0.0005 | <0.001 | | 2.5% |  |
| Fracture of wrist and hand | 190.404 | 0.301 | 0.293 | 0.001 | | 0.048 | 0.0643 | | | 0.0005 | <0.001 | | 2.5% |  |
| Fracture of femur | 201.806 | 0.138 | 0.136 | 0.001 | | 0.001 | 0.0643 | | | 0.0006 | <0.001 | | 2.5% |  |
| eBMD | 354.250 | 2E-19 | <0.001 | -0.002 | | 0.047 | 0.0475 | | | 0.0008 | <0.001 | | 98.5% |  |
| Forearm BMD | 180.5625 | 0.293 | 0.287 | -0.011 | | 0.032 | 0.0592 | | | 0.0164 | <0.001 | | 96.9% |  |
| Femoral neck BMD | 195.380 | 0.048 | 0.049 | -0.001 | | 0.643 | 0.0560 | | | 0.0037 | <0.001 | | 3.1% |  |
| Lumbar spine BMD | 177.365 | 0.209 | 0.063 | -0.003 | | 0.313 | 0.0557 | | | 0.0038 | <0.001 | | 2.8% |  |

Table S4. Mendelian randomization results for causal effect of schizophrenia on FN and LS BMD

| Outcomes | Method | nSNP | beta (95% CI) | P |
| --- | --- | --- | --- | --- |
| FN BMD | IVW | 165 | 0.001 (-0.021, 0.023) | 0.932 |
|  | WM | 165 | -0.003(-0.034, 0.029) | 0.867 |
|  | MR Egger | 165 | 0.018 (-0.058, 0.095) | 0.640 |
|  | Penalised WM | 165 | -0.003(-0.034, 0.028) | 0.849 |
|  | RAPS | 165 | 0.001 (-0.022, 0.025) | 0.901 |
|  | MR LASSO | 160 | -0.003(-0.023, 0.017) | 0.808 |
| LS BMD | IVW | 164 | 0.001(-0.023, 0.026) | 0.935 |
|  | WM | 164 | 0.003(-0.034, 0.039) | 0.878 |
|  | MR Egger | 164 | 0.044(-0.043, 0.130) | 0.323 |
|  | Penalised WM | 164 | 0.003(-0.032, 0.038) | 0.863 |
|  | RAPS | 164 | 0.003(-0.023, 0.030) | 0.796 |
|  | MR LASSO | 162 | 0.006(-0.018, 0.030) | 0.624 |

IVW, inverse-variance weighted; WM, Weighted median.

Table S5. Results of MVMR analyses of the causal effect of schizophrenia on fracture and BMD adjusting for BMI.

| Exposure | Outcomes | nSNP | beta | Se | P |
| --- | --- | --- | --- | --- | --- |
| schizophrenia | Fracture of skull and facial bones | 87 | -0.001 | 0.0002 | 0.006 |
| BMI |  | 500 | 0.001 | 0.0004 | 0.189 |
| schizophrenia | Fracture of femur | 87 | -0.001 | 0.0003 | 0.035 |
| BMI |  | 500 | 0.001 | 0.0005 | 0.934 |
| schizophrenia | eBMD | 87 | 0.022 | 0.008 | 0.009 |
| BMI |  | 499 | 0.125 | 0.017 | 4e-14 |
